# Construction and Validation of an Immune Cell Signature Score to Evaluate Prognosis and Therapeutic Efficacy in Hepatocellular Carcinoma

**DOI:** 10.3389/fgene.2021.741226

**Published:** 2021-09-27

**Authors:** Linfeng Xu, Xingxing Jian, Zhenhao Liu, Jingjing Zhao, Siwen Zhang, Yong Lin, Lu Xie

**Affiliations:** ^1^School of Medical Instrument and Food Engineering, University of Shanghai for Science and Technology, Shanghai, China; ^2^Shanghai Center for Bioinformation Technology, Shanghai Institute for Biomedical and Pharmaceutical Technologies, Shanghai, China; ^3^Bioinformatics Center, National Clinical Research Center for Geriatric Disorders, Xiangya Hospital, Central South University, Changsha, China; ^4^College of Food Science and Technology, Shanghai Ocean University, Shanghai, China

**Keywords:** hepatocellular carcinoma, tumor immune microenvironment, immune cell signature, ICSscore, prognostic stratification, therapeutic evaluation

## Abstract

**Background:** Hepatocellular carcinoma (HCC) is the most common primary liver malignancy with high morbidity and mortality worldwide. Tumor immune microenvironment (TIME) plays a pivotal role in the outcome and treatment of HCC. However, the effect of immune cell signatures (ICSs) representing the characteristics of TIME on the prognosis and therapeutic benefit of HCC patients remains to be further studied.

**Materials and methods:** In total, the gene expression profiles of 1,447 HCC patients from several databases, i.e., The Cancer Genome Atlas (TCGA), International Cancer Genome Consortium, and Gene Expression Omnibus, were obtained and applied. Based on a comprehensive collection of marker genes, 182 ICSs were evaluated by single sample gene set enrichment analysis. Then, by performing univariate and multivariate Cox analysis and random forest modeling, four significant signatures were selected to fit an immune cell signature score (ICSscore).

**Results:** In this study, an ICSscore-based prognostic model was constructed to stratify HCC patients into high-risk and low-risk groups in the TCGA-LIHC cohort, which was successfully validated in two independent cohorts. Moreover, the ICSscore values were found to positively correlate with the current American Joint Committee on Cancer staging system, indicating that ICSscore could act as a comparable biomarker for HCC risk stratification. In addition, when setting the four ICSs and ICSscores as features, the classifiers can significantly distinguish treatment-responding and non-responding samples in HCC. Also, in melanoma and breast cancer, the unified ICSscore could verify samples with therapeutic benefits.

**Conclusion:** Overall, we simplified the tedious ICS to develop the ICSscore, which can be applied successfully for prognostic stratification and therapeutic evaluation in HCC. This study provides an insight into the therapeutic predictive efficacy of prognostic ICS, and a novel ICSscore was constructed to allow future expanded application.

## Introduction

Hepatocellular carcinoma (HCC) accounts for 75 to 85% of primary liver cancer, and is the sixth most common and fourth fatal malignancy globally, with 1- and 3-year survival rates of 20 and 5%, respectively, and a median survival of 8 months (Olsen et al., 2010; Bray et al., 2018). About two-thirds of patients with HCC are frequently diagnosed at advanced stages, being characterized by an aggressive clinical course (Llovet et al., 2016). Although multiple clinical strategies can be applied for HCC treatment, including surgical resection, liver transplantation, radiofrequency ablation, and chemotherapy, the efficacy is limited by high recurrence rate (Bruix and Sherman, 2011; Kuhlmann and Blum, 2013; Heimbach et al., 2018). Currently, the tumor–node–metastasis (TNM) system is still the gold standard for risk stratification of HCC patients (Liu et al., 2016). However, the recurrence and survival for HCC patients vary widely within each stage grouping (Park et al., 2020).

Emerging evidences showed that the tumor immune microenvironment (TIME) plays a key role in the tumor progression, recurrence, and metastasis (Nishida and Kudo, 2017; Kurebayashi et al., 2018). The differences in the composition and abundance of tumor-infiltrating lymphocytes (TILs), such as T cells, macrophages, dendritic cells, and associated fibroblasts, have been reported to influence the prognosis of HCC patients in different ways (Tang et al., 2019). For example, CD45RO+ memory T lymphocyte infiltration leads to a favorable clinical outcome in solid tumors, such as colorectal, gastric, and esophageal cancer, implicating that it is a valuable biomarker for prognostic prediction for human solid malignances (Gabrielson et al., 2016; Hu and Wang, 2017). Further understanding of TIME would provide more advanced prognostic and therapeutic biomarkers for HCC patients (Fu et al., 2019; Zhang et al., 2019). However, only a small number of TILs can be assessed, and the accuracy of applying TILs in predicting prognosis and treatment responding was still limited (Garnelo et al., 2017).

In this study, based on a comprehensive collection of marker genes attached to immune cell signatures (ICSs) from literatures, several HCC transcriptomic datasets were applied to quantify the ICS by single sample gene set enrichment analysis (ssGSEA). Subsequently, after performing univariate and multivariate Cox analysis and random forest modeling, four significant ICSs associated with prognosis were identified to construct an immune cell signature score (ICSscore). In several independent cohorts, the ICSscore was successfully validated to be associated with risk stratification of HCC patients, including tumor vs. normal samples, early- vs. advanced-staging samples, and treatment-responding vs. non-responding samples. Also, the unified ICSscore was validated successfully in other solid tumors, e.g., melanoma and breast cancer.

## Materials and Methods

### Dataset Acquisition and Preprocessing

In this study, several gene expression datasets and the available clinical information of HCC were collected from several databases, including The Cancer Genome Atlas (TCGA), Gene Expression Omnibus, and International Cancer Genome Consortium (ICGC). Therein, in the microarray datasets (GSE14520, GSE96792, GSE109211, and GSE104580), we extracted the probe expression (log2 intensity) and probe annotation, respectively. When a gene was mapped by multiple probes, the expression of the gene was represented by the median of the multiple probes. In the RNA-seq datasets (TCGA-LIHC and ICGC LIRI-JP), we took the read counts to log2-transformation for normalization (Lian et al., 2018). In order to make the gene expression profiling comparable between different platforms, we then normalized with the scale method by using the limma package in R (Wang et al., 2021). Patients with follow-up time 0 or without follow-up were excluded from datasets. The available clinical characteristics of these samples are summarized in Supplementary Table S1.

The HCC datasets (GSE96792 and GSE109211) that received sorafenib treatment were obtained to assess the risk score in treatment-responding or non-responding patients. The HCC dataset (GSE104580) was used to predict therapeutic efficacy of transcatheter arterial chemoembolization (TACE). In addition, the breast cancer and malignant melanoma datasets (GSE20181 and GSE91061) were also downloaded to evaluate risk score and therapeutic effect (Riaz et al., 2017).

### Immune Cell Signatures and Normalized Enrichment Score

In this study, a comprehensive collection of marker genes marked to 184 ICSs was referred from a literature (Wang S. et al., 2020), in which these ICSs and the corresponding marker genes were collected from diverse resources, including previous studies and database. To be specific, 25 signatures were collected from Bindea et al. (2013), 68 signatures were collected from the study of Wolf et al. (2014), 17 signatures were downloaded from the ImmPort database (Bhattacharya et al., 2014), 24 cell signatures were collected from the study of Miao et al. (2020), and 22, 10, and 10 signatures were collected from CIBERSORT (Newman et al., 2015), MCPcounter (Becht et al., 2016; R package, version 1.2.0) and imsig (Nirmal et al., 2018; Rpackage, version 1.1.3), respectively.

To quantify the 184 ICSs in each sample by a normalized enrichment analysis, the ssGSEA was implemented based on the gene expression matrix by using R package GSVA (version 1.36.3; Hänzelmann et al., 2013). Based on the expression of those given genes marked to each ICS, the ssGSEA produces an enrichment fraction, which represents the absolute enrichment degree in each sample. More detailed marked gene sets are listed in Supplementary Table S2. In this study, due to the lack of some marker genes in the transcriptomic profiles, only 182 ICSs were evaluated for subsequent analysis.

### Construction of Immune Cell Signature Score

Since some ICSs with low variance may harm the convergence of hazard ratio (HR), and HR can be adjusted by magnifying the variance of some ICSs, we tried to increase the variance by scaling up ICS 10-fold or 100-fold for subsequent analysis. Based on the quantitative enrichment matrix of the ICS above, we first performed the univariable Cox proportional hazards regression analysis. 26 ICSs were selected with a significance of less than 0.01. Subsequently, a random forest algorithm (R package randomForestSRC, version 2.10.1) was used to narrow down feature selection (Breiman, 2001), in which we set the number of the nsplit as 100 in the variable hunting function (Ishwaran, 2015). The variable importance (VIMP) was used to measure the variation of the random forest model’s prediction error rate. We selected the ICS with the VIMP of higher than 0.01. Here, only four ICSs were retained for subsequent analysis, i.e., CSR_Activated_15701700, CHANG_CORE_SERUM_RESPONSE_UP, Type_1_T_helper_cell, and TREM1_data.

On the basis of the four selected ICSs, multivariable Cox proportional hazards regression analysis was performed, and an ICSscore was constructed based on the quantitative enrichment matrix of the ICS and the corresponding regression coefficients as follows:


$$I\,C\,S\,s\,c\,o\,r\,e=\sum_{i=1}^{4}\beta_{i}*I\,C\,S_{i}$$

Where *ICS*_*i*_ denotes the *i*th ICS and β_*i*_ represents the coefficient of *ICS*_*i*_ obtained from multivariate Cox regression analysis.

In this study, the ICSscore of each sample was calculated by the above formula. In each dataset, those patients were divided into high-risk or low-risk groups based on the median ICSscores in their respective datasets, in order to avoid the batch effect among the different datasets, especially RNA sequencing and microarray.

### Comparison of Immune Cell Signature Score-Based Prognostic Model

Three published prognostic models (Wang Y. et al., 2020; Zhang et al., 2020; Liu P. et al., 2021) regarding HCC were taken to compare our model constructed in this study. The risk scores were calculated for each model, respectively. The differences in continuous score *p*-values and concordance index (C-index) from the univariate Cox analysis were compared, respectively.

### Identification of Differentially Expressed Genes

According to the list of marker genes attached to the 184 ICS, we selected those genes attached to the four selected ICS. Here, a total of 435 unique genes were extracted. Subsequently, by using the R package limma (version 3.44.3), those genes with differential abundance were identified, which met the thresholds of absolute value of log2 fold change greater than 1 and the *p*-value less than 0.05 (Ritchie et al., 2015).

### Machine Learning Classifier Algorithm

XGBoost is an optimization algorithm of gradient boosting decision tree, which is to gather many classification and regression tree models together to form a strong classifier (Jiang et al., 2021). To construct the classifier that could predict responders and non-responders in sorafenib treatment and TACE treatment, we applied the XGBoost algorithm (Python 3.8.3, package XGBoost version 1.3.0).

### Statistical Analysis

In this study, all statistical analyses were implemented in R software (version 4.0.3). The Kaplan–Meier survival curve was visualized by using gsurvplot function implemented in the R package survminer (version 0.4.8) and log-rank test was used to compare the overall survival (OS), progression-free interval (PFI), disease-free interval (DFI), and disease-specific survival (DSS) between the different groups. Univariate Cox regression analysis was used to determine the significant features associated with OS, PFI, DFI, and DSS by calculating HR, 95% confidence interval (CI), and *p*-value between the different groups. Multivariate Cox regression analysis was used to assess the confounding risk score by several significant features. Receiver operating characteristic (ROC) analysis was used to evaluate the accuracy of prognostic model by using the R package survivalROC (version 1.0.3). The boxplot was visualized by using the R package ggpubr (version 0.4.0) and the nomogram and calibration plots were visualized by using the R package rms (version 6.0-1). Subgroup analysis was performed by the coxph function implemented in the R package survival (version 3.2-7) and the forest plot was generated by using the R package forestplot (version 1.10.1).

## Results

### An Immune Cell Signature Score Was Constructed to Significantly Stratify Hepatocellular Carcinoma Patients

Here, 347 HCC samples with OS information in the TCGA-LIHC cohort were used as training dataset for prognostic model construction. First, based on the gene expression profiles and a list of genes marked to ICSs, only 182 ICS were able to be quantitatively evaluated. Subsequently, the evaluated ICSs were used to perform univariate Cox regression analysis, and 26 ICSs were selected with a *p*-value of less than 0.01 (Supplementary Table S3). To further narrow down features, we carried out dimension reduction analysis by using random forest algorithm, and four ICSs were identified with the VIMP of larger than 0.01, including CHANG_CORE_SERUM_RESPONSE_UP, CSR_Activated_15701700, TREM1_data, and Type_1_T_helper_ cell (Supplementary Figure S1A). Eventually, the four selected ICSs were applied to construct a multivariate Cox prognostic model, in which an ICSscore was formulated based on the quantitative ICSs and their corresponding coefficients. The associations between the four ICSs and OS are illustrated in Supplementary Figure S1B, and the C-index of the prognostic model reached 0.70.

In order to examine whether ICSscore was an independent prognostic factor in each subgroup, ICSscore was applied to separately perform univariate Cox analysis in different subgroups of the TCGA-LIGC cohort, such as age, gender, American Joint Committee on Cancer (AJCC) stage, and vascular tumor cell type. As illustrated in Supplementary Figure S2, except for the AJCC stage IV, ICSscore could stratify HCC patients in the other subgroups significantly. However, in HCC patients of AJCC stage IV, the insignificance of ICSscore to stratify HCC patients may be due to the small sample size.

**FIGURE 1:**
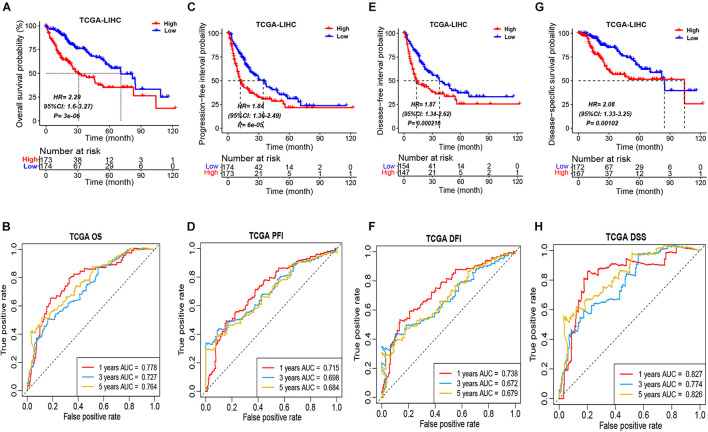
Prognostic stratification of ICSscore in the TCGA-LIHC cohort. Patients were assigned to high-level and low-level groups by setting the median ICSscore as the cutoff. **(A)** The overall survival probability of high-level and low-level groups was evaluated (log-rank test, *p* = 3E-06). **(B)** The survival AUCs of 1-, 3-, and 5-year overall survival rate, respectively, were 0.778, 0.727, and 0.784. **(C)** The progression-free interval probability of high-level and low-level groups was evaluated (log-rank test, *p* = 6E-05). **(D)** The survival AUCs of 1-, 3-, and 5-year progression-free interval rate, respectively, were 0.715, 0.698, and 0.684. **(E)** The disease-free interval probability of high-level and low-level groups was evaluated (log-rank test, *p* = 0.000216). **(F)** The survival AUCs of 1-, 3-, and 5-year disease-free interval rate, respectively, were 0.738, 0.672, and 0.679. **(G)** The disease-specific survival probability of high-level and low-level groups was evaluated (log-rank test, *p* = 0.00102). **(H)** The survival AUCs of 1-, 3-, and 5-year disease-specific survival rate, respectively, were 0.827, 0.774, and 0.826.

According to the median ICSscore in the TCGA-LIHC cohort, the patients can be divided into high-risk and low-risk groups. As shown in Figure 1A, the patients in the high-risk group showed significantly poorer OS than those in the low-risk group, indicating that high-level ICSscore is associated with worse outcomes. Furthermore, to assess the sensitivity and specificity of the ICSscore-based prognostic model, we performed ROC analysis. The area under curve (AUC) achieved 0.778, 0.727, and 0.764, respectively, at the 1-, 3-, and 5-year OS rate (Figure 1B), suggesting that the ICSscore-based prognostic model has a good prediction performance.

Moreover, the differences in PFI, DFI, and DSS between the high-risk and low-risk groups in the TCGA-LIHC cohort were also compared, respectively. Consistently, the patients in the high-risk group all showed obviously poorer PFI (Figure 1C), DFI (Figure 1E), and DSS (Figure 1G). Meanwhile, through performing the ROC analysis on PFI, DFI, and DSS, the comparable AUCs are shown in Figures 1D,F,H. These implied that the ICSscore constructed by the four significant ICS can significantly stratify HCC patients.

To provide a clinically applicable risk assessment model for predicting the prognosis of HCC patients, a nomogram that integrated ICSscore and AJCC staging was constructed in the TCGA-LIHC cohort (Supplementary Figure S3A). According to the nomogram illustrated in this study, a combination of ICSscore and AJCC stage of a HCC patient can be calculated to predict the 1-, 3-, and 5-year OS for an individual. In addition, as illustrated in Supplementary Figures S3B–D, the calibration curves at the 1-, 3- and 5-year OS for an individual all fit well to the ideal curves. Noteworthy, we found that the ICSscore contributed to the most risk points when compared with the AJCC staging, suggesting that ICSscore would make a greater predictive contribution.

### The Validation of the Immune Cell Signature Score-Based Prognostic Model

**FIGURE 2:**
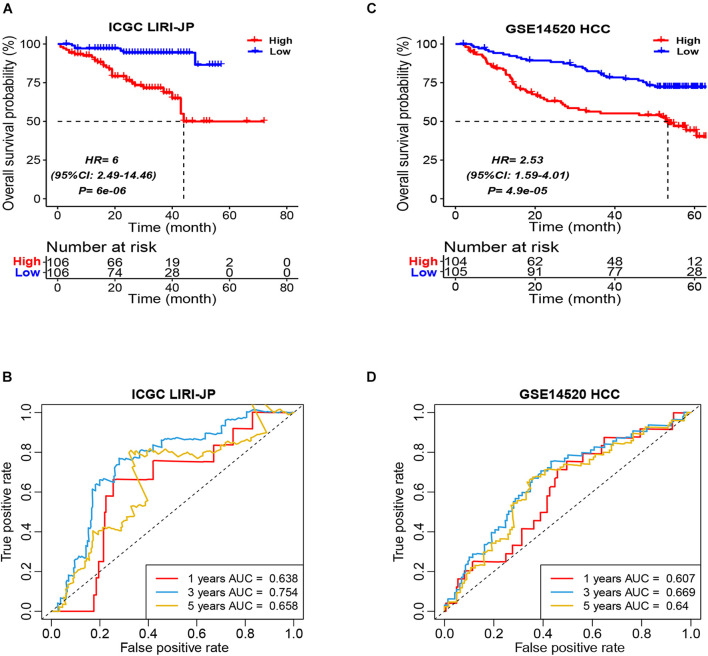
Prognostic stratification of ICSscore in the ICGC LIRI-JP and GSE14520 HCC cohort. Patients were assigned to high-level and low-level groups by setting the respective median ICSscore as the cutoff. **(A)** The overall survival probability of high-level and low-level groups was evaluated in the ICGC LIRI-JP cohort (log-rank test, *p* = 6E-06). **(B)** The survival AUCs of 1-, 3-, and 5-year overall survival rate, respectively, were 0.638, 0.754, and 0.658. **(C)** The overall survival probability of high-level and low-level groups was evaluated in the GSE14520 HCC cohort (log-rank test, *p* = 4.9E-05). **(D)** The survival AUCs of 1-, 3-, and 5-year overall survival rate, respectively, were 0.607, 0.669, and 0.64.

In order to validate the robustness of the ICSscore-based prognostic model trained in the TCGA-LIHC cohort, two independent datasets (i.e., ICGC LIRI-JP and GSE14520 HCC) were applied, respectively. Similarly, in the two validation cohorts, according to their individual median ICSscore, we divided patients into two groups, i.e., high-ICSscore and low-ICSscore groups. Consistent with the findings above, the high-level ICSscore group showed significantly poorer prognostic outcomes (Figures 2A,C). Meanwhile, the ROCs were also analyzed in the two validation cohorts. The AUC of the prognostic model was 0.638, 0.754, and 0.658, respectively, at 1-, 3-, and 5-year survival rates in the IGCG LIRI-JP cohort (Figure 2B), and the AUC was 0.607, 0.669, and 0.640, respectively, at 1-, 3-, and 5-year survival rates in the GSE14520 HCC cohort (Figure 2D). These results demonstrated that the ICSscore can be used to stratify HCC patients and predict prognosis.

In addition, in light of the ICSscore in different subgroups of the ICGC LIRI-JP cohort, we separately carried out univariate Cox analysis, such as age, gender, TNM stage, virus, and vein invasion. Except for those subgroups with small sample sizes, ICSscore did stratify significantly HCC patients (Supplementary Figure S4), suggesting that ICSscore was a robust biomarker to stratify patients in the different subgroups.

Furthermore, in the ICGC LIRI-JP cohort, a nomogram that integrated ICSscore and TNM staging was constructed as well (Supplementary Figure S5A). Compared with TNM staging, we also observed that the ICSscore contributed to the most risk points, demonstrating that the ICSscore can make a greater predictive contribution. Meanwhile, the calibration curves at the 1-, 3- and 5-year OS were all found to be close to the ideal curves (Supplementary Figures S5B–D).

### The Comparison of Risk Stratification and Predictive Ability of Immune Cell Signature Score as a Feature

To compare the risk stratification and predictive ability of ICSscore, we calculated the continuous prognostic risk scores and concordance index (C-index) by performing univariate Cox analysis. Compared with age, gender, AJCC stage, and invasion (Tables 1, 2), the C-index of the ICSscore was higher and the *p*-value of the ICSscore was lower, indicating ICSscore to be a good predictor.

**TABLE 1:** Comparison of the *p*-value and C-index derived from the univariate Cox model in the TCGA-LIHC cohort.

Signatures	*p*-value	C-index
ICSscore	1.82E-13	0.700
Baohui_Zhang_2020	8.89E-10	0.694
Yu_Wang_2020	3.08E-13	0.690
Peng_Liu_2021	9.56E-09	0.640
Age	0.1881	0.508
Gender	0.2614	0.507
AJCC_STAGE	1.52E-05	0.609
Vascular_tumor_cell_type	0.123	0.533

**TABLE 2:** Comparison of the *p*-value and C-index derived from the univariate Cox model in the ICGC-JP cohort.

Signatures	*p*-value	C-index
ICSscore	9.44E-05	0.711
Baohui_Zhang_2020	7.75E-5	0.707
Yu_Wang_2020	0.0001582	0.680
Peng_Liu_2021	0.00285	0.671
Age	0.3973	0.536
Gender	0.07557	0.566
TNM_STAGE	0.0001536	0.704
VEIN_INVASION	0.004346	0.615

In addition, three published prognostic models (Wang Y. et al., 2020; Zhang et al., 2020; Liu P. et al., 2021) regarding HCC were used to compare our ICSscore-based prognostic model constructed in this study. The continuous prognostic risk scores were calculated for each model by performing univariate Cox analysis, respectively, in TCGA-LIHC and ICGC-JP cohorts. As shown in Tables 1, 2, these differences in *p*-values and C-index were compared, suggesting that our ICSscore-based prognostic model has a preferrable predictive ability.

### Differential Marker Genes in the Four Immune Cell Signatures Formulating Immune Cell Signature Score

**FIGURE 3:**
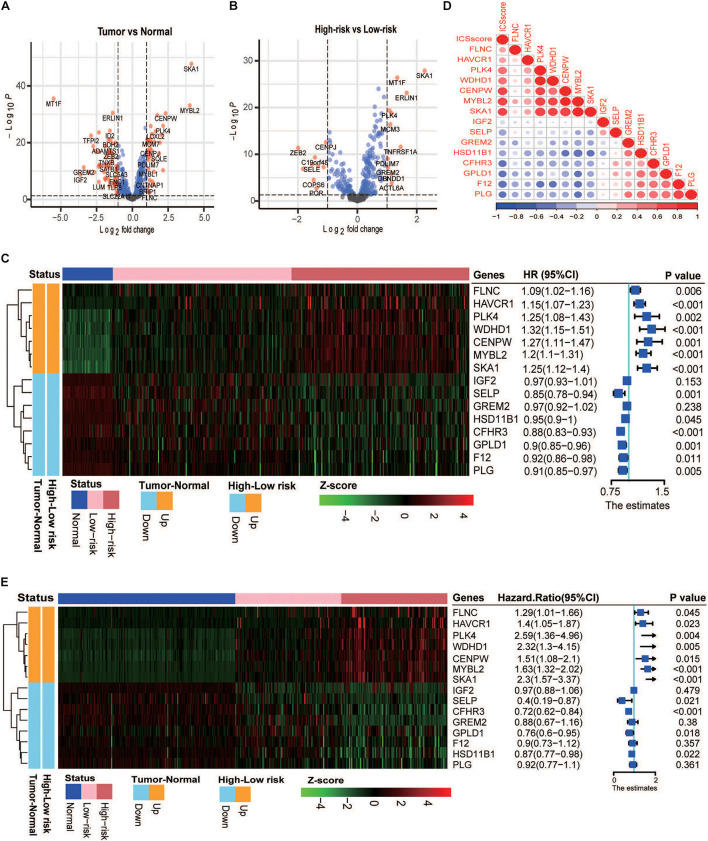
Analysis of differential gene expression in the TCGA-LIHC and ICGC-JP cohort. **(A)** Volcano plot presents the differentially expressed genes (DEGs) between the tumor and normal samples. **(B)** Volcano plot presents the DEGs between the high-risk and low-risk samples. **(C)** Left: heatmap shows the scaled abundance of 15 DEGs among normal, low-risk, and high-risk samples. Right: forest plot denotes the association between the DEGs and overall survival. The HR, 95% CI, and *p*-value were determined by univariate Cox regression analysis in the TCGA-LIHC cohort. **(D)** Correlations between expression levels of 15 genes and the ICSscore values. **(E)** Left: heatmap shows the scaled abundance of 15 DEGs among normal, low-risk, and high-risk samples. Right: forest plot denotes the association between the DEGs and overall survival. The HR, 95% CI, and *p*-value were determined by univariate Cox regression analysis in the ICGC-JP cohort.

To explore the underlying reason of ICSscore in risk assessment and prognostic prediction, 435 marker genes attached to the four significant ICS formulating ICSscore were investigated. In the TCGA-LIHC cohort, the gene expression matrix from 347 tumor samples and 49 normal samples was used for subsequent differential analysis. First, between the tumor and normal samples, a total of 97 differentially expressed genes (DEGs) were identified, including 36 up-regulated genes and 61 down-regulated genes (Figure 3A). Similarly, between the high-risk and low-risk samples as distinguished above, we obtained 21 DEGs, including 11 up-regulated genes and 10 down-regulated genes (Figure 3B), which were speculated to make more contribution to differential ICSscore evaluation. Thus, we performed GO enrichment and several significant biological processes were obtained (Supplementary Figure S6), such as activated T-cell proliferation, positive regulation of wound healing, and regulation of activated T-cell proliferation. In addition, of these 21 genes, 15 were found to be the same as those between tumor and normal samples (Figure 3C). Notably, sequentially comparing the normal samples, the low-risk samples, and the high-risk samples, we found that the abundance of genes *FLNC*, *HAVCR1*, *PLK4*, *WDHD1*, *CENPW*, *MYBL2*, and *SKA1* increased, while genes *IGF2*, *SELP*, *GREM2*, *HSD11B1*, *CFHR3*, *GPLD1*, *F12*, and *PLG* decreased. Furthermore, univariate analysis of these genes showed that the upregulated genes were detrimental to HCC prognosis, while the down-regulated genes were beneficial (Figure 3C). Indeed, most of these genes have been reported as prognostic biomarkers or suggested as novel therapeutic targets for HCC. For example, the overexpression of genes *CENPW*, *MYBL2*, and *SKA1* is associated with poor prognosis in HCC, while the loss of gene *HSD11B1* indicates poor prognosis in HCC (Frau et al., 2011; Chen et al., 2018; Zhou et al., 2020). Moreover, we focused on the correlations between the ICSscore value and expression levels of 15 genes. As illustrated in Figure 3D, the up-regulated genes were positively correlated with the ICSscore, while the down-regulated genes were negatively correlated. Moreover, in the ICGC-JP cohort, as shown in Figure 3E, the abundance alteration of the above 15 genes and their association with prognosis were observed to be consistent.

### Evaluation and Prediction of Disease Malignancy and Molecular Target Therapy Benefit in Hepatocellular Carcinoma by Immune Cell Signature Score

**FIGURE 4:**
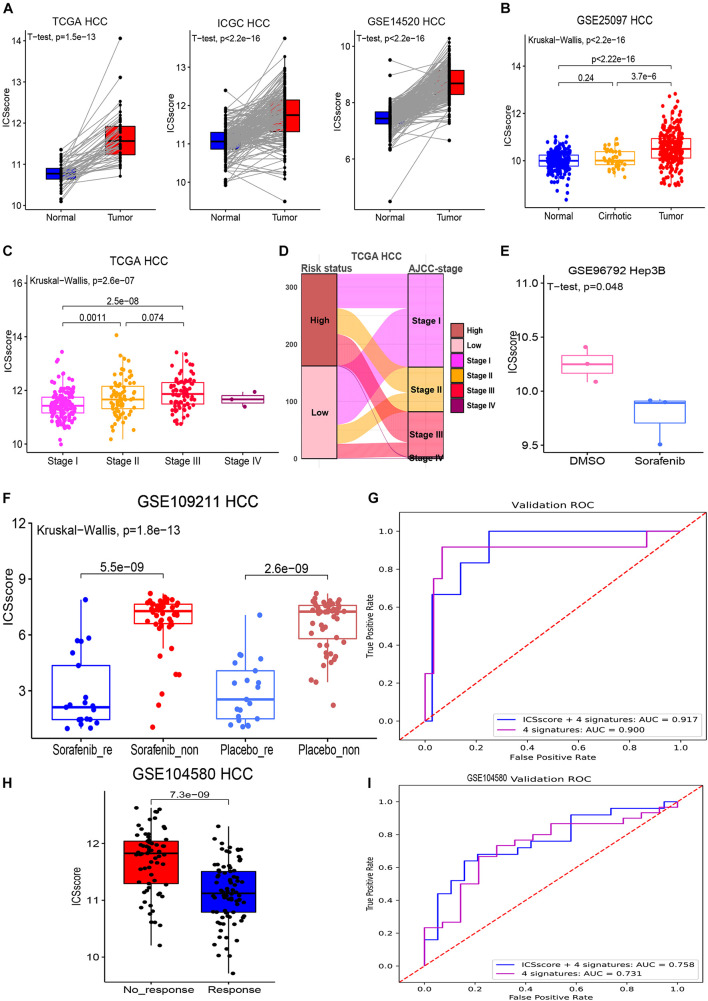
Evaluation and prediction of disease malignancy and molecular target therapy benefit in HCC by ICSscore. **(A)** Pairwise comparison of the ICSscore between normal and tumor samples in three cohorts, i.e., TCGA-LIHC (*t*-test *p* = 1.5E-13), ICGC LIRI-JP (*t*-test *p* < 2.2E-13), and GSE14520 HCC (*t*-test *p* < 2.2E-13). **(B)** Boxplot illustrates the differences of the ICSscore values among normal, cirrhotic, and tumor samples in the GSE25097 HCC cohort. **(C)** Boxplot illustrates the differences of the ICSscore values among different AJCC staging of the TCGA-LIHC cohort. **(D)** Sankey plot shows the mapping between high or low ICSscore and AJCC staging of the TCGA-LIHC cohort. **(E)** Boxplot shows the ICSscore values of Hep3B cell line treated with sorafenib or DMSO in the GSE96792 cohort. **(F)** Boxplot illustrates the ICSscore values of responded or non-responded HCC patients treated with sorafenib or placebo in the GSE109211 cohort. **(G)** ROC curve of the XGBoost algorithm for predicting the responding and non-responding patients in the GSE109211 cohort. **(H)** Boxplot illustrates the ICSscore values of responding or non-responding HCC patients treated with chemotherapy in the GSE104580 cohort. **(I)** ROC curve of the XGBoost algorithm for predicting the responding and non-responding patients in the GSE104580 cohort.

In order to verify whether the ICSscore evaluation was consistent with other risk stratification methods, several HCC cohorts were compared. First, as illustrated in Figure 4A, in the three HCC cohorts, tumor samples all exhibited strikingly higher ICSscore values when compared with the paired normal samples. In the GSE25097 HCC cohort, we also found that the tumor samples showed the highest ICSscore values, while the normal samples showed relatively low ICSscore values, although there was no significant difference between the normal samples and cirrhotic samples (Figure 4B). These results indicate a significant increase in ICSscore when hepatocytes develop into tumors.

Recently, the eighth edition staging system of the AJCC was released for HCC stratification (Park et al., 2020). In the TCGA-LIHC cohort, after excluding three stage IV samples, the stage III samples showed the highest ICSscore, followed by stage II samples, and the stage I samples exhibited the lowest ICSscore, indicating that the ICSscore was positively correlated with the current risk stratification system (Figure 4C). As expected, we found that most advanced-staging patients (stage III and stage IV) were assigned into the high-risk group, while more early-staging patients (stage I and II) were designated into the low-risk group (Figure 4D), implying that the ICSscore could act as a comparable marker for HCC risk stratification. At the same time, we also observed that some early-staging patients were assigned to the high-risk group, while late-staging patients were assigned to the low-risk group, suggesting that ICSscore may be used as a supplement to compromise AJCC-staging risk stratification errors. Indeed, studies have reported significant differences in recurrence and survival for HCC patients within each AJCC stage grouping.

Furthermore, we explored whether ICSscore could be used as a marker to evaluate therapeutic efficacy. Sorafenib is the only Food and Drug Administration-approved first-line targeted agent for the treatment of advanced HCC, but its impact on patient survival is limited depending on the pathogenetic conditions (Bruix et al., 2017). Here, the gene expression profiles (GSE96792) from the Hep3B cell line treated with sorafenib or DMSO was obtained to evaluate the ICSscore, respectively. As a result, we did observe lower ICSscore in those Hep3B treated with sorafenib compared with those Hep3B treated with DMSO (Figure 4E), suggesting that the ICSscore may be used to reflect therapeutic efficacy. Subsequently, we further tested the ICSscore in a clinical trial on sorafenib (GSE1090211), and those patients who responded to sorafenib showed much lower ICSscore than those who had no response to sorafenib (Figure metricconverterProductID4F4F). Interestingly, in those patients treated with placebo, we also observed that responding patients exhibited much lower ICSscore than non-responding patients (Figure metricconverterProductID4F4F). These results implied that the ICSscore could be used to predict the therapeutic benefit. Therefore, when setting the four ICSs and ICSscores as features, two classification models were separately constructed to predict responding and non-responding samples, in which 70% of the data in the GSE109211 cohort was taken as the training set, and 30% as the validation set. The AUCs for predicting treatment responding were achieved at 0.917 and 0.900, respectively, (Figure 4G). Similarly, those HCC patients who received chemotherapy in GSE104580 cohorts were examined as well. Consistently, compared with those HCC patients who had no response to chemotherapy, much lower ICSscores were observed in those patients who responded to chemotherapy (Figure 4H). Also, when setting the four ICSs and ICSscores as features to build classification models, the AUCs for predicting treatment-responding patients were achieved at 0.758 and 0.733, respectively, (Figure 4I). These findings implied that the ICSscore may be used as an indicator for prediction of treatment responding in HCC.

### Evaluation and Prediction of Chemotherapy and Immunotherapy Benefit in Other Tumors by Immune Cell Signature Score

**FIGURE 5:**
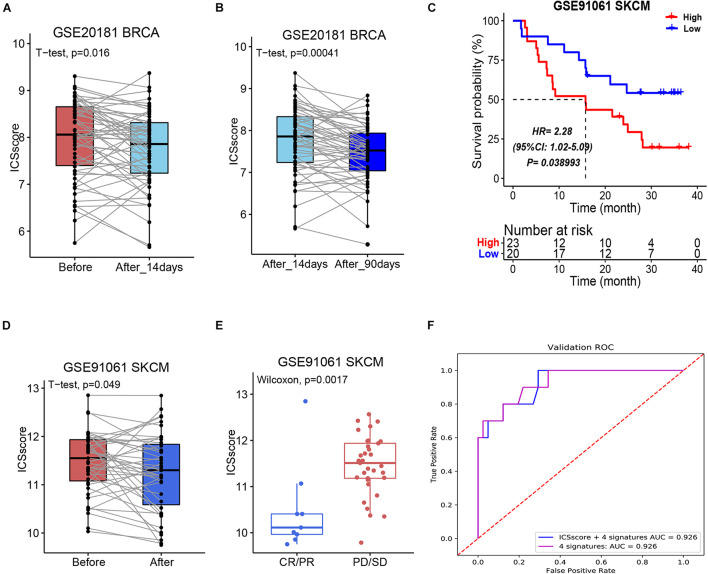
Evaluation and prediction of chemotherapy and immunotherapy benefit in other tumors by ICSscore. **(A,B)** Pairwise comparison of the ICSscore in patients before and after chemotherapy in the GSE20181 BRCA cohort. **(C)** Kaplan–Meier curves of overall survival according to low- and high-ICSscore groups in the GSE91061 SKCM cohort. **(D)** Pairwise comparison of the ICSscore in patients before and after immunotherapy in the GSE91061 SKCM cohort. **(E)** Boxplot illustrates the ICSscore of patients with immunotherapy response in the GSE91061 SKCM cohort. **(F)** ROC curve of the XGBoost algorithm for predicting the therapeutic effects in the GSE91061 SKCM cohort.

As most patients with a high-level ICSscore displayed poorer prognosis and low therapeutic benefit than those with a low-level ICSscore in HCC, we explored whether the ICSscore could predict therapeutic benefit in other tumors. For this investigation, a cohort of breast cancer patients with chemotherapy information (GSE20181) were first applied to calculate the unified ICSscore value for each patient on the basis of transcriptomic profiles and marker genes. When comparing the pairwise ICSscore before and after treatment with adjuvant chemotherapy, we found that the patients’ ICSscore significantly decreased after 14-day adjuvant chemotherapy (Figure 5A), and it decreased further after 90-day adjuvant chemotherapy (Figure 5B). That is to say, further adjuvant chemotherapy led to a gradual decrease of ICSscore, suggesting a gradual therapeutic benefit. This suggests that the ICSscore could be used to monitor therapeutic efficacy in breast cancer.

More recently, the strategy for immune checkpoints, PD-1 and PD-L1, has become an immune therapy with amazing survival benefit (Liu C. et al., 2021). Unfortunately, the effectiveness of immune checkpoint therapy is limited because only a small number of patients respond to the therapy. Here, a cohort of melanoma patients who received anti-PD1 and anti-CTLA4 therapy (GSE91016) were also applied to evaluate the ICSscore application. By setting the mean ICSscore value as the cutoff, these patients were classified into high-ICSscore and low-ICSscore groups. Similarly, the high-ICSscore group exhibited significantly poorer OS (Figure 5C). In addition, by pairwise comparing the ICSscore between patients before and after immunotherapy, we observed that the patients’ ICSscore was significantly decreased after receiving immunotherapy (Figure 5D). The result implied that lower ICSscore values can be used to distinguish those patients who benefit from immunotherapy. Indeed, as shown in Figure 5E, the patients with CR/PR presented lower ICSscore than those with PD/SD. Subsequently, setting the four ICSs and ICSscores as features, we constructed two classification models to predict whether the patients received therapeutic benefit. The AUCs in the training set were all achieved at 0.926 (Figure 5F). Thus, the ICSscore value may be used as a predictive biomarker for immunotherapeutic benefit in melanoma.

## Discussion

A large number of studies have demonstrated that the TILs are associated with tumor progression and patient prognosis (Zheng et al., 2017; Ding et al., 2018; Lu et al., 2019). In the present study, on the basis of a comprehensive collection of marker genes, 182 ICSs associated with TIME were evaluated and applied. Here, an ICSscore formulated by the four-best prognosis-related ICS was constructed, which was validated successfully to predict prognosis and therapeutic benefit in HCC. Indeed, the four ICSs have significant associations to the tumor immune system, and a dozen marker genes attached to the four signatures have been reported to predict prognosis. For example, CHANG_CORE_SERUM_RESPONSE_UP was reported to correlate with wound healing, with elevated expression of angiogenic genes, a high proliferation rate, and a Th2 cell bias to the adaptive immune infiltrate. TREM1_data was marked by the only gene TREM1, which triggers phagocyte secretion of pro-inflammatory chemokines and cytokines. However, the specific biological roles of these ICSs remain to be further explored. In particular, the fitted ICSscore was found to be positively correlated with the risk level of HCC patients, but negatively correlated with the therapeutic efficacy. That is to say, the fitted ICSscore not only can be used to predict prognosis, but also can be used as an effective biomarker to evaluate therapeutic benefit and monitor treatment efficacy.

Sorafenib has been considered the standard of care for patients with advanced unresectable HCC since 2007 (Abdelgalil et al., 2019). It is an important step to detect patients who would potentially benefit from sorafenib treatment. Here, we proved that the ICSscores were significantly reduced in sorafenib-responding HCC patients, indicating that the ICSscore may be a biomarker for predicting the response to sorafenib in HCC patients. Moreover, chemotherapy is one of the most important treatment modalities for advanced HCC. Significantly decreased ICSscores were observed in chemotherapy-responding HCC patients, indicating that the ICSscore can also be used as a marker for predicting the response to chemotherapy in HCC patients. Even so, due to the limitations of therapeutic datasets with regard to HCC, more real-world datasets are needed to further verify our findings and improve the ICSscore, especially those datasets using different treatments, such as immunotherapy. Similarly, gradually decreased ICSscore values were observed in breast cancer patients receiving chemotherapy for 14 days and 90 days, and significantly declined ICSscore values were found in melanoma patients with partial or complete remission after immunotherapy. These results imply that the ICSscore evaluation may be applied in pan cancer therapy supervision.

In recent years, immunotherapy exhibited promising therapeutic effects for advanced HCC, although only a few patients benefited from immunotherapy (Johnston and Khakoo, 2019; Riley et al., 2019; Zongyi and Xiaowu, 2020). Further research is needed to select effective biomarkers for patients who might benefit from immunotherapy. To our pleasure, the ICSscore evaluation may be used as a biomarker to distinguish patients who would respond to immunotherapy.

There were also some limitations in this study. Firstly, given that the large number of HCC patients used in this study came from different platforms, there may be significant batch effects in our cohort. Secondly, a series of ICSs were marked here, but only a few were used to construct the ICSscore. Thirdly, due to the limitation of datasets with treatment information, it is necessary to further testify and optimize ICSscore as a marker for immunotherapy in HCC, and even a broad spectrum of pan cancer.

## Conclusion

Overall, we simplified the tedious ICSs to develop ICSscore, which can be applied successfully in prognostic stratification and therapeutic evaluation in HCC. Also, in melanoma and breast cancer, the unified ICSscore was validated to distinguish the samples with therapeutic benefits. This study provides a novel insight into the prognosis and therapeutic efficacy of ICS. ICSscore may be a potential marker for therapeutic efficacy in HCC, and even a broad spectrum of pan cancer.

## Data Availability Statement

The original contributions presented in the study are included in the article/Supplementary Material; further inquiries can be directed to the corresponding author/s.

## Ethics Statement

All procedures were in accordance with the ethical standards of the institutional and national committee on human experimentation and with the Helsinki Declaration (revised in 2013). There was no interaction with patients directly, as we acquired data from online public datasets.

## Author Contributions

LX, YL, LFX, and XJ conceived and designed this study. LFX and XJ were responsible for dataset collection, bioinformatics analysis, wrote the draft manuscript, and results interpretation. ZL, JZ, and SZ contributed to data analysis and discussion. LX revised the manuscript. The author(s) read and approved the final manuscript. All authors contributed to the article and approved the submitted version.

## Conflict of Interest

The authors declare that the research was conducted in the absence of any commercial or financial relationships that could be construed as a potential conflict of interest.

## Publisher’s Note

All claims expressed in this article are solely those of the authors and do not necessarily represent those of their affiliated organizations, or those of the publisher, the editors and the reviewers. Any product that may be evaluated in this article, or claim that may be made by its manufacturer, is not guaranteed or endorsed by the publisher.

## Supplementary Material

Supplementary Figure S1 Feature selection in TCGA-LIHC cohort. **(A)** The trend of the error rate changes with the depth of the treeand the variable importance ranking from random survival forest. **(B)** Theforest plot of the associations between the four selectedimmune cell signatures and overall survival in the TCGA cohort. The HR, 95% CI, and *p*-value weredetermined by multivariate Cox regression analysis.

Supplementary Figure S2 The forest plot shows the association between ICSscore and overall survival in the subgroups of TCGA cohort.

Supplementary Figure S3 Nomogram and Calibration developed in the TCGA-LIHC cohort. **(A)** A nomogram used to predict 1-year, 3-year, and 5-year overall survival probabilities. **(B)** Calibration curve of the overall survival of HCC patients for 1-, 3-, and 5-years.

Supplementary Figure S4 **|** The forest plot shows the association between ICSscore and overall survival in the subgroups of ICGC HCC cohort.

Supplementary Figure S5 **|** Nomogram and calibration developed in the ICGC HCC cohort. **(A)** A nomogram used to predict 1-year, 3-year, and 5-year overall survival probabilities. **(B)** Calibration curve of the overall survival of HCC patients for 1-, 3-, and 5-years.

Supplementary Figure S6 **|** The GO enrichment analysis based on the differentially expressed genes between high-risk and low-risk groups.

Supplementary Table S1 **|** The clinical characteristics of liver cancer patients in the three cohorts.

Supplementary Table S2 **|** 184 immune cell signatures and their corresponding genes.

Supplementary Table S3 **|** The significant immune cell signatures by performing univariate Cox regression analyses.
