# Supplementary material for: Construction and Validation of an Immune Cell Signature Score to Evaluate Prognosis and Therapeutic Efficacy in Hepatocellular Carcinoma
Source: Front Genet. 2021 Sep 27;12:741226. doi: 10.3389/fgene.2021.741226 (PMC8503558; doi:10.3389/fgene.2021.741226)
Supplement: Supplementary Figure S1 — Feature selection in TCGA-LIHC cohort. (A) The trend of the error rate changes with the depth of the treeand the variable importance ranking from random survival forest. (B) Theforest plot of the associations between the four selectedimmune cell signatures and overall survival in the TCGA cohort. The HR, 95% CI, and p-value weredetermined by multivariate Cox regression analysis. [file Presentation_1.zip › Supplement Materials/Supplement Figures legend.docx]

**Figure S1: Feature selection in TCGA-LIHC cohort. (A)** The trend of the error rate changes with the depth of the tree and the variable importance ranking from random survival forest. **(B)** The forest plot of the associations between the 4 selected immune cell signatures and overall survival in the TCGA cohort. The HR, 95% CI and P-value were determined by multivariate Cox regression analysis.

**Figure S2:** The forest plot shows the association between ICSscore and overall survival in the subgroups of TCGA cohort.

**Figure S3: Nomogram and Calibration developed in the TCGA-LIHC cohort.** **(A)** A nomogram used to predict 1-year, 3-year, and 5-year overall survival probabilities. **(B)** Calibration curve of the overall survival of HCC patients for 1-, 3- and 5-year.

**Figure S4:** The forest plot shows the association between ICSscore and overall survival in the subgroups of ICGC HCC cohort.

**Figure S5: Nomogram and Calibration developed in the ICGC HCC cohort.** **(A)** A nomogram used to predict 1-year, 3-year, and 5-year overall survival probabilities. **(B)** Calibration curve of the overall survival of HCC patients for 1-, 3- and 5-year.

**Figure S6:** The GO enrichment analysis based on the differentially expressed genes between high-risk and low-risk groups.
