# Supplementary figures and images for: Construction and Validation of an Immune Cell Signature Score to Evaluate Prognosis and Therapeutic Efficacy in Hepatocellular Carcinoma
Source: Front Genet. 2021 Sep 27;12:741226. doi: 10.3389/fgene.2021.741226 (PMC8503558; doi:10.3389/fgene.2021.741226)

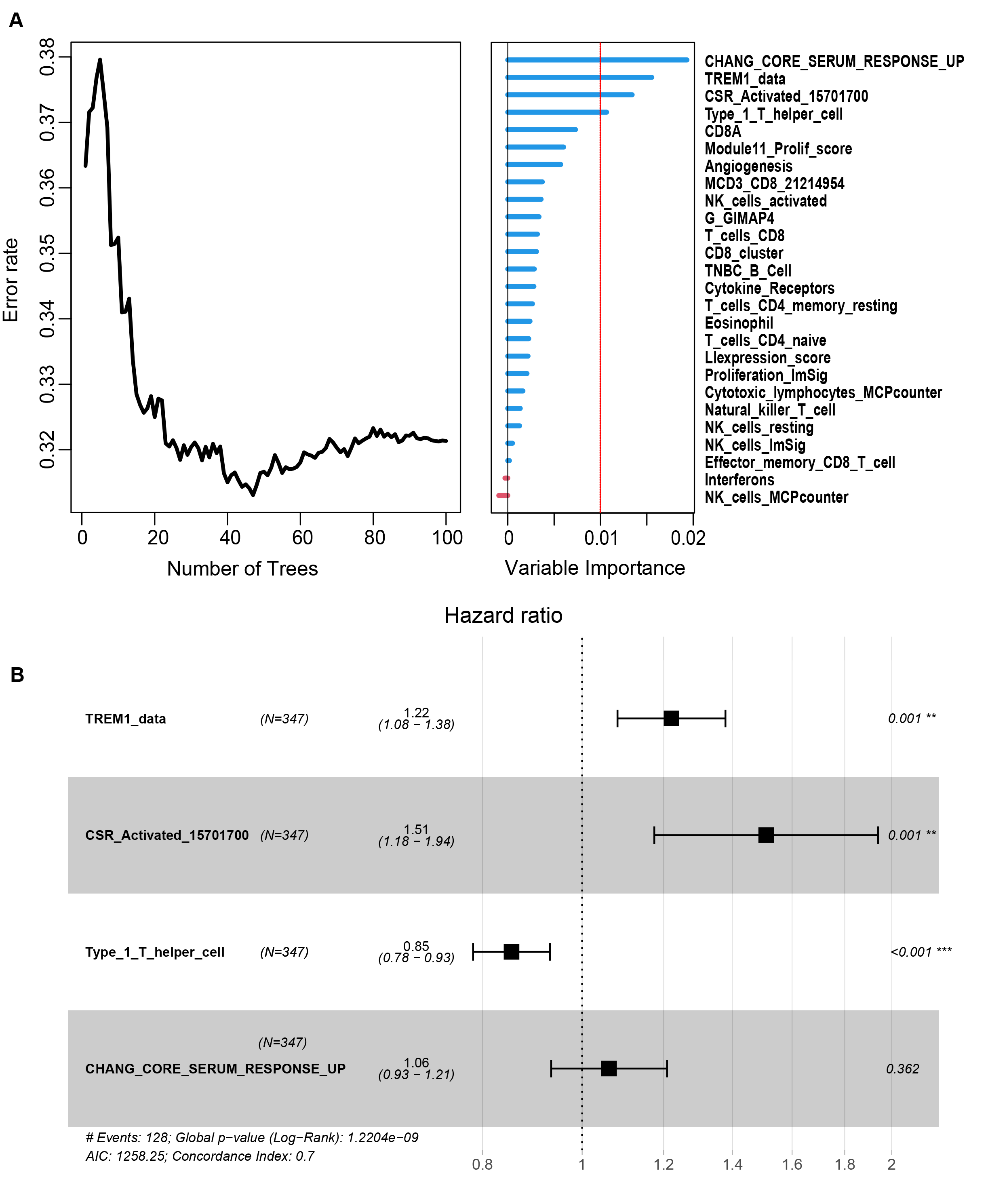

Supplement: Supplementary Figure S1 — Feature selection in TCGA-LIHC cohort. (A) The trend of the error rate changes with the depth of the treeand the variable importance ranking from random survival forest. (B) Theforest plot of the associations between the four selectedimmune cell signatures and overall survival in the TCGA cohort. The HR, 95% CI, and p-value weredetermined by multivariate Cox regression analysis. [file Presentation_1.zip › Supplement Materials/Figures-S1.tif]

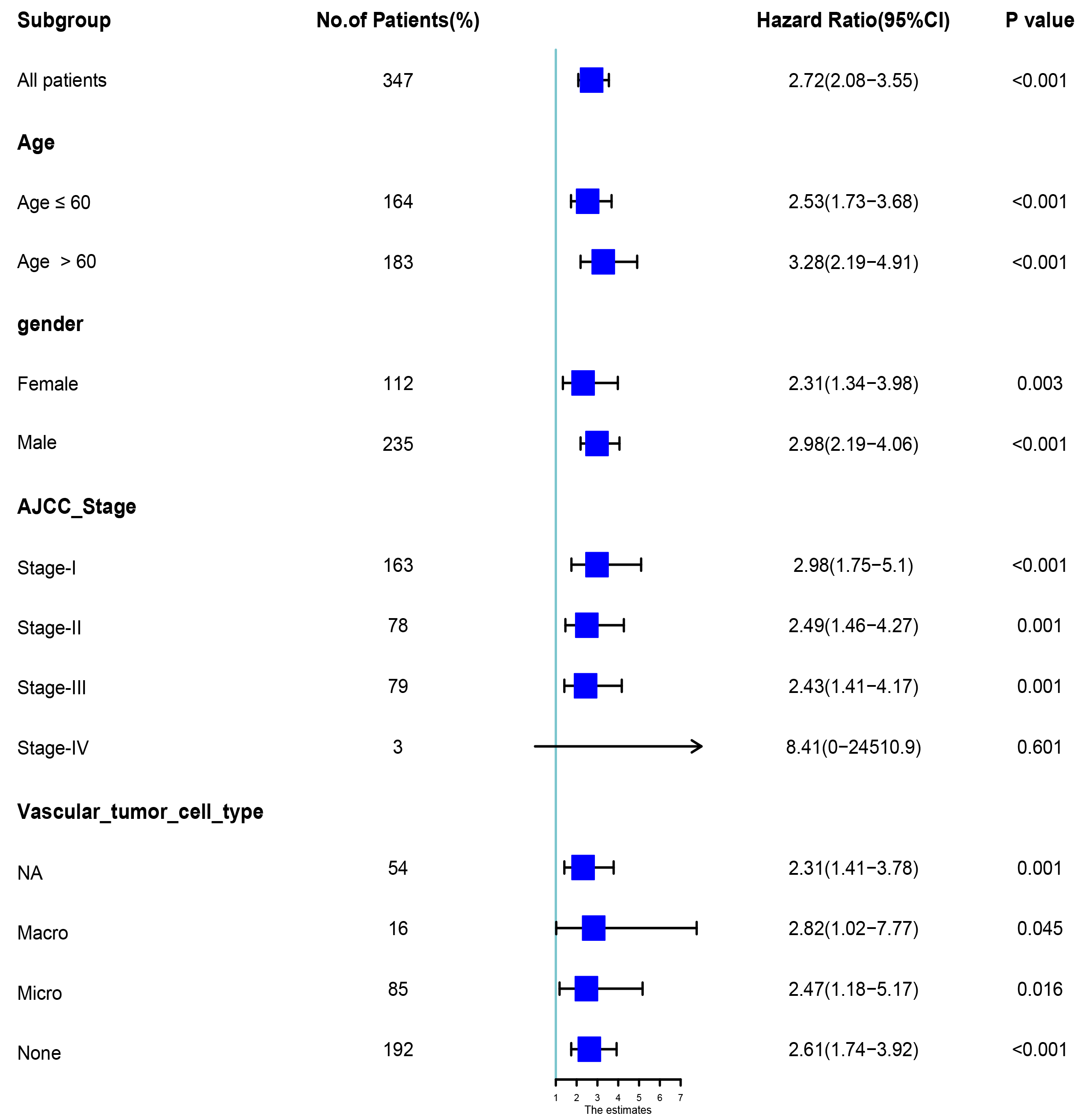

Supplement: Supplementary Figure S1 — Feature selection in TCGA-LIHC cohort. (A) The trend of the error rate changes with the depth of the treeand the variable importance ranking from random survival forest. (B) Theforest plot of the associations between the four selectedimmune cell signatures and overall survival in the TCGA cohort. The HR, 95% CI, and p-value weredetermined by multivariate Cox regression analysis. [file Presentation_1.zip › Supplement Materials/Figures-S2.tif]

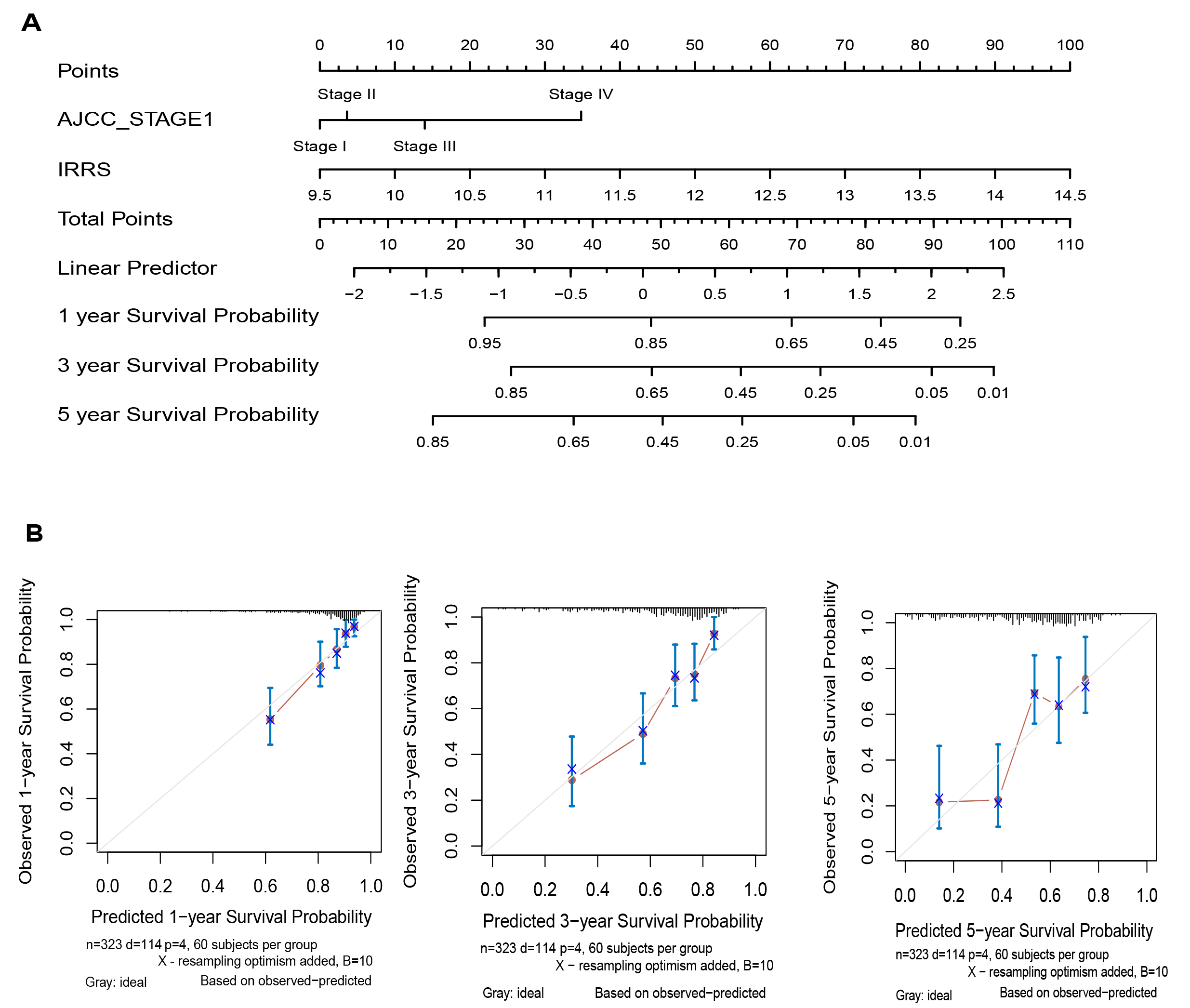

Supplement: Supplementary Figure S1 — Feature selection in TCGA-LIHC cohort. (A) The trend of the error rate changes with the depth of the treeand the variable importance ranking from random survival forest. (B) Theforest plot of the associations between the four selectedimmune cell signatures and overall survival in the TCGA cohort. The HR, 95% CI, and p-value weredetermined by multivariate Cox regression analysis. [file Presentation_1.zip › Supplement Materials/Figures-S3.tif]

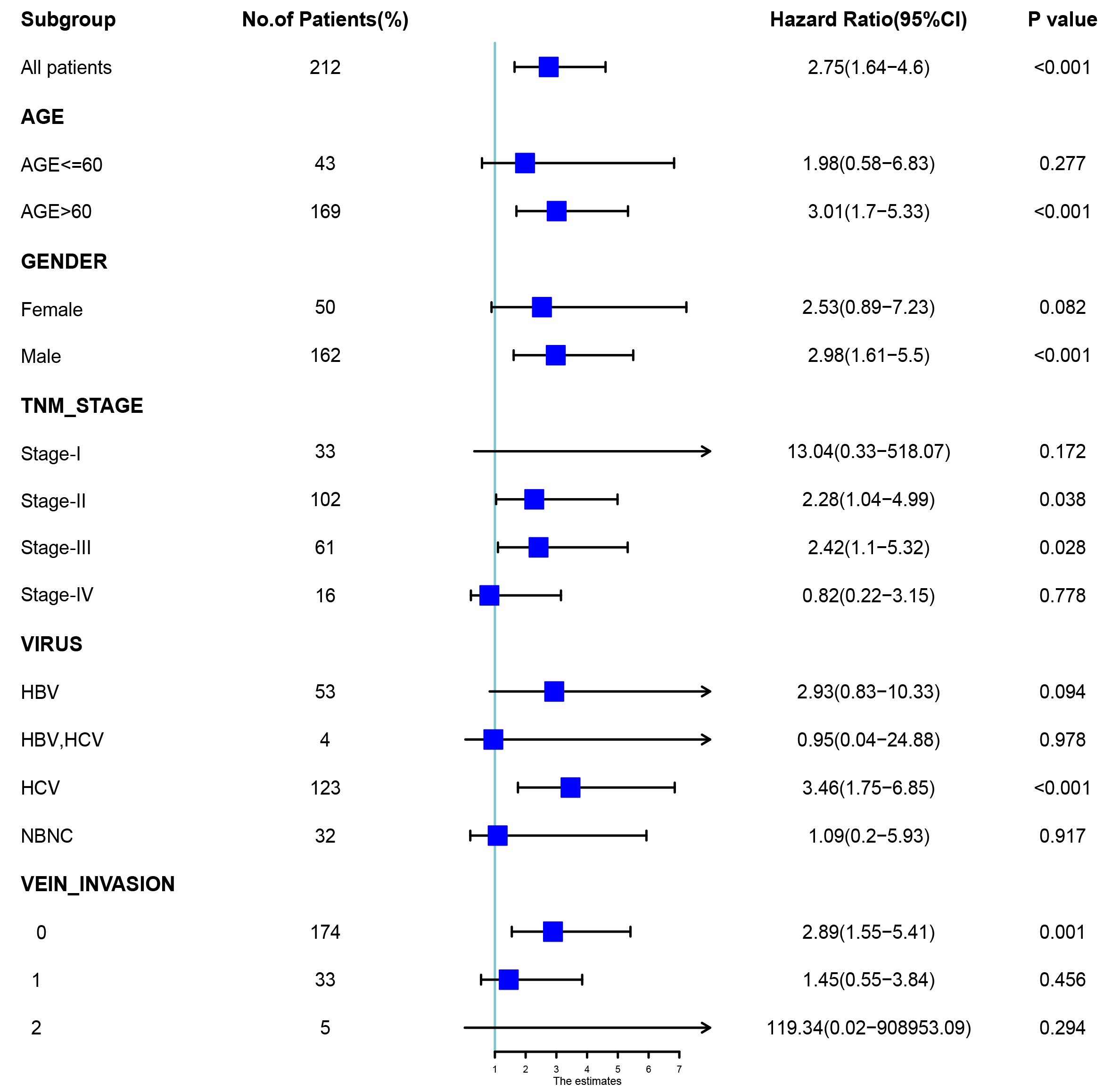

Supplement: Supplementary Figure S1 — Feature selection in TCGA-LIHC cohort. (A) The trend of the error rate changes with the depth of the treeand the variable importance ranking from random survival forest. (B) Theforest plot of the associations between the four selectedimmune cell signatures and overall survival in the TCGA cohort. The HR, 95% CI, and p-value weredetermined by multivariate Cox regression analysis. [file Presentation_1.zip › Supplement Materials/Figures-S4.tif]

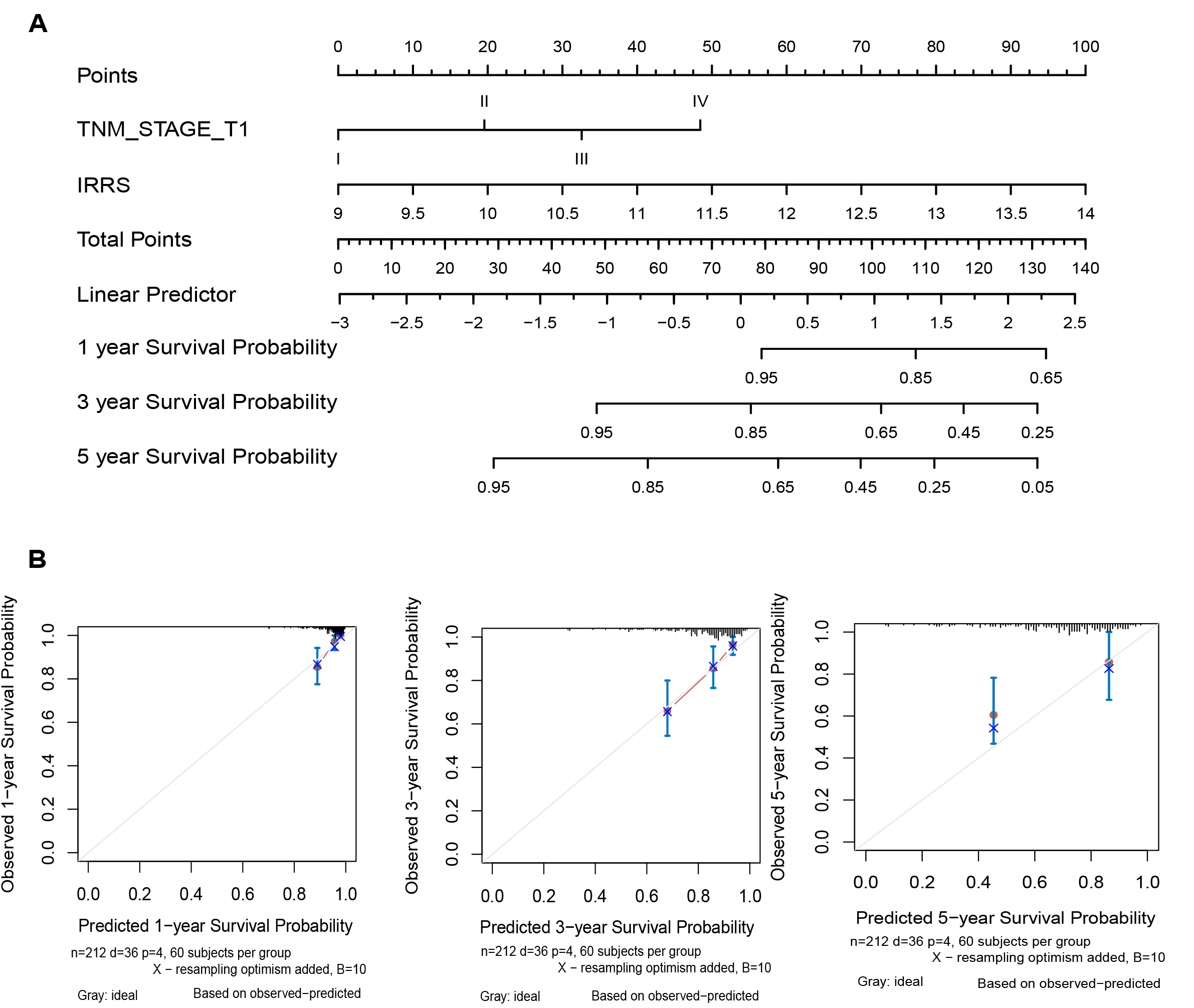

Supplement: Supplementary Figure S1 — Feature selection in TCGA-LIHC cohort. (A) The trend of the error rate changes with the depth of the treeand the variable importance ranking from random survival forest. (B) Theforest plot of the associations between the four selectedimmune cell signatures and overall survival in the TCGA cohort. The HR, 95% CI, and p-value weredetermined by multivariate Cox regression analysis. [file Presentation_1.zip › Supplement Materials/Figures-S5.tif]

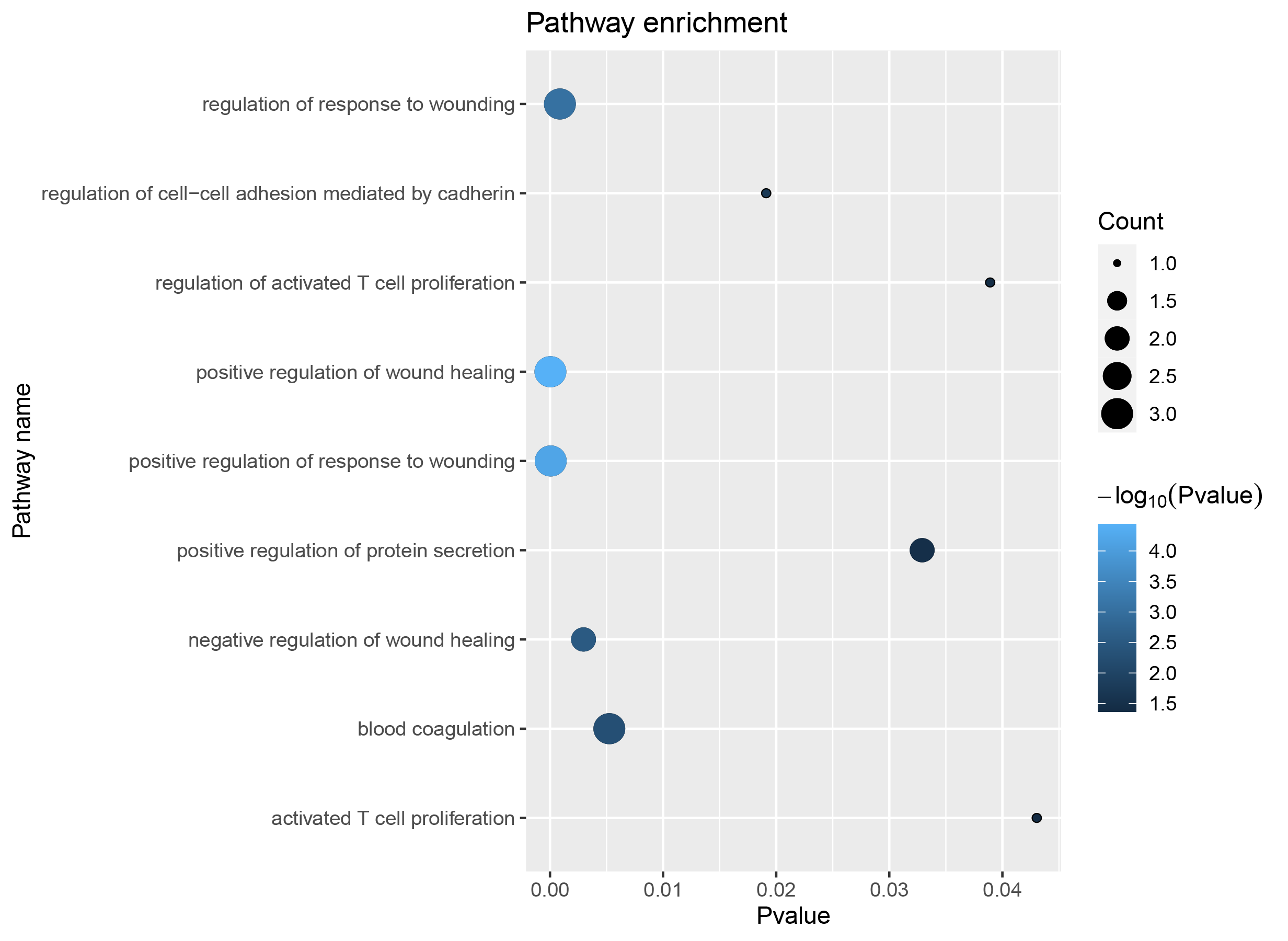

Supplement: Supplementary Figure S1 — Feature selection in TCGA-LIHC cohort. (A) The trend of the error rate changes with the depth of the treeand the variable importance ranking from random survival forest. (B) Theforest plot of the associations between the four selectedimmune cell signatures and overall survival in the TCGA cohort. The HR, 95% CI, and p-value weredetermined by multivariate Cox regression analysis. [file Presentation_1.zip › Supplement Materials/Figures-S6.tif]
